# Dyslipidemia in Kidney Disorders: Perspectives on Mitochondria Homeostasis and Therapeutic Opportunities

**DOI:** 10.3389/fphys.2020.01050

**Published:** 2020-09-03

**Authors:** Pei-Hui Lin, Pu Duann

**Affiliations:** ^1^Department of Surgery, Davis Heart and Lung Research Institute, The Ohio State University, Columbus, OH, United States; ^2^Research and Development, Salem Veteran Affairs Medical Center, Salem, VA, United States

**Keywords:** fatty acid β-oxidation, energy metabolism, lipotoxicity, podocyte, proximal tubule cells, oxidative stress, homeostasis, fibrosis

## Abstract

To excrete body nitrogen waste and regulate electrolyte and fluid balance, the kidney has developed into an energy factory with only second to the heart in mitochondrial content in the body to meet the high-energy demand and regulate homeostasis. Energy supply from the renal mitochondria majorly depends on lipid metabolism, with programed enzyme systems in fatty acid β-oxidation and Krebs cycle. Renal mitochondria integrate several metabolic pathways, including AMPK/PGC-1α, PPARs, and CD36 signaling to maintain energy homeostasis for dynamic and static requirements. The pathobiology of several kidney disorders, including diabetic nephropathy, acute and chronic kidney injuries, has been primarily linked to impaired mitochondrial bioenergetics. Such homeostatic disruption in turn stimulates a pathological adaptation, with mitochondrial enzyme system reprograming possibly leading to dyslipidemia. However, this alteration, while rescuing oncotic pressure deficit secondary to albuminuria and dissipating edematous disorder, also imposes an ominous lipotoxic consequence. Reprograming of lipid metabolism in kidney injury is essential to preserve the integrity of kidney mitochondria, thereby preventing massive collateral damage including excessive autophagy and chronic inflammation. Here, we review dyslipidemia in kidney disorders and the most recent advances on targeting mitochondrial energy metabolism as a therapeutic strategy to restrict renal lipotoxicity, achieve salutary anti-edematous effects, and restore mitochondrial homeostasis.

## Introduction

The kidney is characterized by a complex anatomy, with millions of nephrons as the functional unit to excrete nitrogen waste and secure fluid homeostasis. The kidney is composed of multiple specialized cell types ensuring vital homeostasis of acid-base and electrolyte balance, blood pressure regulation, nutrient reabsorption, and hormone secretion (Hoenig and Zeidel, 2014; Duann and Lin, 2017; Yu et al., 2019). Therefore, it is one of the most metabolically active organs other than heart and skeletal muscle, with proximal tubules presenting a very high density of mitochondria required for energy consumption (Meyer et al., 1997; Wang et al., 2000). For example, the human proximal convoluted tubules (S1 and S2 combined) contain abundant large mitochondria, which occupy about 16.3% cell volume (Møller and Skriver, 1985). Notably, the mature nephron comprises distinct segments, each utilizing metabolic pathways to varying degrees depending on the specific function (Cargill and Sims-Lucas, 2020).

Complete oxidation of fatty acids (FAs), which are high-energy substrates, to CO_2_ and H_2_O gives rise to roughly 9 Kcal/g fat, while only 4 Kcal/g are generated from carbohydrates or proteins. The heart possesses metabolic flexibility and powerful catabolic capacity to use various energy substrates, mainly FAs (40–60%) and glucose (20–40%), for ATP production (Karwi et al., 2018). Instead, most proximal tubule epithelial cells (PTEC) have low metabolic flexibility toward glycolysis and rely on FAs as energy source at baseline (Bonventre and Yang, 2011). This was shown by early *in vivo* studies measuring ATP synthesis by tracking isotope-labeled FAs with NMR in rat kidney, which indicated that FAs are a preferred fuel (Freeman et al., 1986). However, PTEC are able to shift to anaerobic glycolysis to produce ATP required for cellular regeneration after ischemic acute kidney injury (AKI; Lan et al., 2016). In mice, glomerular podocytes display much lower mitochondrial density than in PTECs and rely primarily on anaerobic glycolysis to maintain glomerular filtration barrier and are relatively insensitive to defect in mitochondrial biogenesis during ischemia damage (Brinkkoetter et al., 2019). Instead, as lipid accumulation is commonly observed in patients with chronic kidney disease, podocytes are rather sensitive to cellular cholesterol-mediated glomerular injury (Merscher et al., 2014).

Mitochondria are pivotal for maintaining the health and function of the metabolically active kidney by providing efficient energy support through the process of oxidative phosphorylation (OXPHOS) and aerobic glycolysis. Several factors such as mitochondria biogenesis, bioenergetics, dynamics, and autophagy regulate the mitochondrial physiology (Duann and Lin, 2017). In addition, mitochondria also contribute to production of reactive oxygen species (ROS) free radicals and transduction of metabolic and stress signals (Galvan et al., 2017; Flemming et al., 2018). Persistent mitochondrial damage is a major source of oxidants. Consequently, mitochondrial fitness translates into body’s general health. Mitochondrial dysfunction is involved in various kidney diseases, such as acute kidney injury (AKI), chronic kidney disease (CKD), diabetic nephropathy (DN), and glomerulonephritis (GN; Duann and Lin, 2017; Eirin et al., 2017; Galvan et al., 2017; Flemming et al., 2018).

Dysregulated lipid metabolism with defective cholesterol/free fatty acid (FFA) metabolism leading to dyslipidemia is common in patients of several kidney diseases, including acute kidney disease, CKD, diabetic kidney disease (DKD), nephrotic syndrome, and uremia (Agrawal et al., 2017; Hager et al., 2017; Gai et al., 2019; Nishi et al., 2019; Nishi and Nangaku, 2019; Jang et al., 2020b), and may contribute to end-stage kidney disease. In this review, we summarize the recent advances in understanding lipid metabolism in the function of kidney mitochondria and the molecular mechanisms related to dyslipidemia during kidney disease progression.

## Basic Lipid Biology

In biological systems, lipids include fats, sterols, phospholipids, and triacylglycerides (TAG). In the cell, lipids have numerous functions: they constitute the cell membrane as a protective barrier; form membranous compartments of intracellular organelles; provide energy source and storage; provide building blocks for hormones; and serve as secondary cellular messengers within body. FAs are carboxylic acids with a long aliphatic tail, which constitute building blocks for other lipids such as TAG and phospholipids.

Within the body, lipid metabolism comprises several inter-dependent pathways for the generation, storage, and transport of lipids, which involves plasma lipoprotein particles [chylomicrons, high density lipoproteins (HDL), low density lipoproteins (LDL), intermediate density lipoproteins (IDL), and very low density lipoproteins (VLDL)] in circulation. Dietary lipids, mainly (95%) TAG, some FFAs, and cholesterol, carried by chylomicrons into circulation, are degraded into FFAs and glycerol by lipoprotein lipase (LPL) activity on the capillaries. These FFAs are taken up by muscle, heart, and adipose and peripheral tissues like kidney; remnants of chylomicrons are subsequently cleared in the liver (Florens et al., 2016; Agrawal et al., 2017; Kronenberg, 2018). FFAs are transported by serum albumin to the liver and periphery and could be stored as TAG in kidney capillaries. Additionally, esterified cholesterol could be stored as a lipid droplet within the kidney.

## Transport of Cellular FFAs

### Lipid Uptake by CD36 in the Kidney

**Figure 1:**
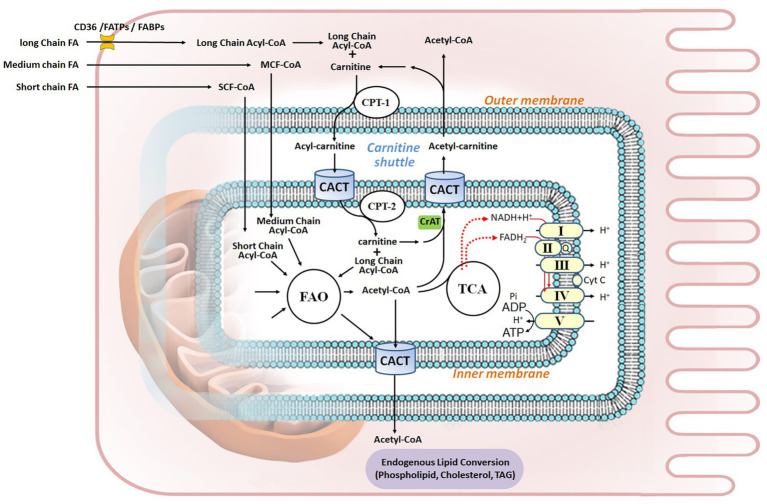
Fatty acid metabolism in renal proximal tubule epithelial cell. Fatty acids (FAs) are the preferred energy substrates for the kidney. Uptake of FAs from capillaries into kidney cells is facilitated by either FAT/CD36 or FABPs and FATPs. In the cytosol, FAs are activated to acyl-CoA, esterified with carnitine, and transported into the mitochondrial matrix through the carnitine shuttle, which is composed of CPT-1, CACT, and CPT-2. Medium-chain FAs and short-chain FAs can permeate the mitochondrial membranes. In the matrix, acyl-CoA undergoes FA β-oxidation (FAO), thereby generating acetyl-CoA to fuel the TCA cycle, as well as FADH_2_ and NADH that serve as electron donors to the five ETC complex for ATP production *via* oxidative phosphorylation. Acetyl-CoA can be shuttled out of mitochondria through carnitine acetyltransferase (CrAT), while it goes through integrated endogenous lipid conversion to form phospholipids, cholesterol, and triacylglycerol (TAG). Q, Coenzyme Q; Cyt C, cytochrome C.

FA uptake from the extracellular milieu is the first step in their utilization. Multiple cell surface lipid transport proteins, such as cluster of differentiation 36 (CD36), scavenger receptor B1 (SR-B1), tissue-specific fatty acid transport proteins (FATPs), and plasma membrane fatty acid-binding protein (FABP_pm_) facilitate cellular FFA uptake (Haunerland and Spener, 2004; Su and Abumrad, 2009; Figure 1).

Long-chain fatty acids (LCFAs, referring to FAs with 12 or longer carbons chains) primarily enter the cell *via* FA transporter CD36 [also known as Fatty acid translocase (FAT) or SR-B2]. CD36 is expressed in multiple cell types and mediates diverse functions, such as lipid uptake, inflammation, ROS production, molecular adhesion, and apoptosis. CD36 is a multifunctional receptor for many ligands, including collagen, native lipoproteins, LCFA, oxidized phospholipids, oxidized LDL, thrombospondin, and apoptotic cells (Yang et al., 2017; Wang and Li, 2019). Several post-translational modifications, including phosphorylation, palmitoylation, ubiquitylation, and glycosylation regulate CD36 stability and dimerization, and correlate its function to myocellular FA uptake (Luiken et al., 2016). In adipocytes, two palmitoyl-acyltransferases (PATs), namely DHHC4/5, modulate CD36 palmitoylation and target it to the plasma membrane lipid rafts, where it mediates FA adsorption and transport (Wang et al., 2019). Interestingly, in addition to the cell surface, CD36 also localizes to the ER, endosomes, and mitochondria (Bonen et al., 2000; Smith et al., 2011). In response to diverse signaling transduction pathways, rapid mobilization of the vesicular transport system mediates dynamic intracellular distribution of CD36 to reprogram energy utilization and control lipid metabolism (Georgiou et al., 2015; Glatz et al., 2016).

In the kidney, CD36 mediates FA uptake and lipid metabolic reprograming and functions (Yang et al., 2017). CD36 is highly expressed in mesangial cells (Ruan et al., 1999), renal proximal (Susztak et al., 2005) and distal tubular epithelial cells (Okamura et al., 2007), podocytes (Hua et al., 2015), microvascular endothelial cells, and interstitial macrophages (Rahman et al., 2008; Kennedy et al., 2013). Transgenic mice with tubular overexpression of CD36 demonstrate tubular-specific accumulation of lipids, TAG, and LCFAs (Kang et al., 2015). Other kidney CD36 substrates include oxidized phospholipids, advanced oxidation protein products (AOPPs; Li et al., 2019), and advanced glycation end products (AGEs), which promote inflammation, ER stress, and renal cells apoptosis and contribute to renal fibrosis (Okamura et al., 2009; Ruggiero et al., 2014; Pennathur et al., 2015).

### Lipid Uptake by Other Transporters in the Kidney: FABPs and FATPs

FA-binding proteins (FABPs) are low molecular weight (14–15 kDa) proteins that transport LCFAs through cell membranes, transport FAs to mitochondria and peroxisomes, and function as chaperones to mediate intracellular transport. Two major FABP isoforms are expressed in human kidneys, the proximal tubule-enriched FABP1 (also known as liver type L-FABP) and the distal tubule-enriched FABP3 (Maatman et al., 1991). Urinary FABP1 level was proposed as a biomarker of acute tubulointerstitial damage (Yamamoto et al., 2007; Pelsers, 2008).

Emerging data also support proximal tubular apical expression of FA transporter-2 (FATP2, encoded by *Slc27a2*) and its role in luminal non-esterified FA (NEFA) reabsorption from glomerular filtrate and NEFA metabolism in mice. Silencing of FATP2 in human renal PTEC *in vitro* leads to increased Oil Red O staining and subsequent apoptosis following FA exposure. Moreover, tubular lipoapoptosis in lipidated albumin-injected mice decreases in *Slc27a2*-deficient mice. These data suggest that luminal NEFA uptake by FATP2 causes proximal tubule lipoapoptosis, which may contribute to tubular atrophy and CKD progression (Khan et al., 2018).

## Fatty Acid Metabolism in Kidney Mitochondria

FA β-oxidation (FAO) may occur in both mitochondria and peroxisomes. While mitochondria majorly oxidize LCFAs, and medium-chain and short-chain FAs (MCFAs and SCFAs, referring to FAs with less than 12 carbons chains), peroxisomes oxidize specific carboxylic acids such as very long-chain FAs (VLCFAs), branched-chain FAs, fatty dicarboxylic acids, and bile acid intermediates (in the liver; Cipolla and Lodhi, 2017). Interestingly, peroxisomal FAO provides alternative metabolism of LCFAs and MCFAs in case of mitochondrial long-chain FAO deficiencies (Violante et al., 2019). Mitochondrial FAO is thus the major pathway for the degradation of FAs to sustain cellular energy homeostasis (Houten et al., 2016). This process includes six tightly-regulated steps: (i) FA esterification to acyl-CoA; (ii) mitochondrial CPT shuttle (or the carnitine shuttle); (iii) the FAO pathway; (iv) the OXPHOS pathway; (v) allosteric control of FAO; and (vi) integrated nutrient metabolism in the kidney (Figure 1).

### Fatty Acid Esterification to acyl-CoA

FAs must be converted to fatty acyl-CoA by cytosolic acyl-CoA synthetases in order to enter mitochondria. Once inside the cell, MCFAs or SCFAs can freely diffuse into mitochondria. However, LCFAs need to be activated to long-chain acyl-CoA (LC acyl-CoA) and esterified with carnitine into LC-acylcarnitine to permeate the outer mitochondrial membrane (OMM) and subsequently be transported into the mitochondrial matrix (Bremer, 1983).

### The Carnitine Shuttle

The carnitine shuttle, mediated by the rate-limiting enzyme carnitine palmitoyltransferase I (CPT-1, on the OMM) and the two inner mitochondrial membrane (IMM) proteins carnitine-acylcarnitine translocase (CACT) and carnitine palmitoyltransferase II (CPT-2), serves to transport the FA moiety into mitochondria. CPT-2 conducts a reverse reaction to convert LC-acylcarnitine back to LC acyl-CoA and carnitine. Carnitine is transported back to the cytoplasm by the same shuttle (Brivet et al., 1999).

### The FAO Pathway

FAO is the process of breaking down a LC acyl-CoA into acetyl-CoA molecules inside the mitochondrial matrix. The term β-oxidation refers to the position of the carbon group being oxidized. The number of acetyl-CoA molecules produced depends on the initial carbon length of the FA. When LC acyl-CoA enters FAO, two carbons are cleaved to generate an acetyl-CoA and an acyl-CoA that is two carbons shorter from each β-oxidation cycle. This process continues until all of the carbons in the FA are turned into acetyl-CoA to fuel the tricarboxylic acid (TCA) cycle and generate ATP. The two redox active coenzymes – the reduced form of nicotinamide adenine dinucleotide (NADH) and the hydroquinone form of flavin adenine dinucleotide (FADH_2_) – produced during each β-oxidation cycle, along with those generated from TCA cycle, are used as electron donors by the electron transport chain (ETC) complex, in the redox reaction that produces ATP (the OXPHOS pathway). LCFA oxidation yields high energy: for instance, 137 ATP are generated from palmitate as opposed to 38 obtained from glucose oxidation (Nsiah-Sefaa and McKenzie, 2016).

### The OXPHOS Pathway

The mitochondrial ETC/OXPHOS respiratory chain contains five complexes. Complexes I–IV transfer electrons (e^−^) and protons (H^+^) across IMM to generate an electrochemical gradient for ATP synthesis in complex V (ATP synthase). Several critical steps regulate this process. The concentration of NAD constitutes the rate-limiting process (Canto et al., 2015; Verdin, 2015). Coenzyme Q10 (CoQ10) is a component of ETC, which shuttles electrons in the respiratory chain. Moreover, the reduced form of CoQ10 is also a potent antioxidant (Ernster and Dallner, 1995; Thomas et al., 1996). CoQ deficiency could cause nephropathies (Ozaltin, 2014) and mutation in ADCK4 (CoQ8B), a protein required for stabilizing CoQ complex in podocyte, is an etiology of steroid-resistant nephrotic syndrome (SRNS or FSGS; Ashraf et al., 2013; Widmeier et al., 2020). Cardiolipin, an IMM phospholipid, plays a central structural role in cristae formation, facilitates ETC supra-complex formation for optimal OXPHOS activity, and serves as a platform to initiate apoptosis (Birk et al., 2014; O’Brien et al., 2015).

### Allosteric Control of FAO

Mitochondrial bioenergetic homeostasis is subjected to allosteric regulation by the ratios of the [Acetyl CoA/CoA], [NADH/NAD^+^], and [FADH_2_/FAD^+^]. Therefore, FAO enzymatic activities are affected by the levels of the metabolic products of their own reactions, and a rise in [Acetyl-CoA/CoA] or [NADH/NAD^+^] leads to feedback inhibition of FAO (Karwi et al., 2019). For example, mice with proximal tubule-specific deletion of carnitine acetyltransferase (CrAT), an enzyme that controls inter-conversion of Acetyl-CoA/CoA and shuttles excess FA products out of the mitochondria, develop mitochondrial dysfunction, cellular apoptosis, and tubular and glomerular fibrosis (Kruger et al., 2019). Interestingly, *de novo* synthesis of NAD^+^, a central metabolic coenzyme/co-substrate involved in cellular energy metabolism, profoundly affects mitochondrial fitness in organ health and injury, including kidney (Hershberger et al., 2017; Katsyuba et al., 2018; Poyan Mehr et al., 2018; Ralto et al., 2020).

### Integrated Nutrient Metabolism in the Kidney

Acetyl-CoA is a critical metabolite derived from catabolism of all major nutrient sources, such as glucose, FAs, and amino acids. Moreover, acetyl-CoA can be diverted from the TCA cycle to synthesize cholesterol, phospholipids, and TAG in the cell (Pietrocola et al., 2015; Shi and Tu, 2015). Proper integration and regulation of energy metabolism during ATP loss or excess are thus key to maintain mitochondrial health during injury and repair in renal pathophysiology (Vamecq et al., 2012; Aon et al., 2014; Fornoni et al., 2014; Szeto, 2017; Jang et al., 2020a).

## Transcriptional, Epigenetic, and Post-Translational Regulation of FAO and Mitochondria Biogenesis

**Figure 2:**
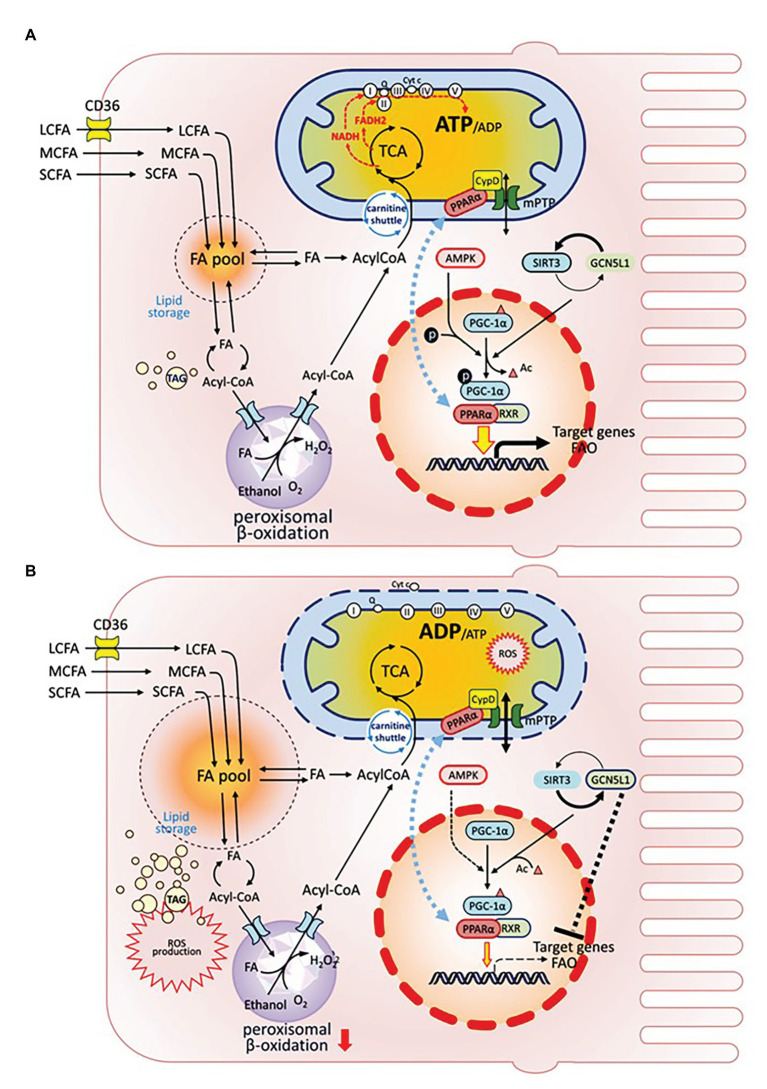
Organelle crosstalk regulates fatty acids metabolism in renal PTEC under healthy or injury/disease states. Intracellular FA metabolism includes catabolic and anabolic pathways. FAs are oxidized either in mitochondria or peroxisome to generate ATP (catabolism), or are stored as global triglyceride pool (anabolism). PGC-1α is the mitochondrial master regulator, which drives mitochondrial biogenesis by co-activating transcriptional factors PPAR-α and RXR to regulate the expression of target genes affecting biogenesis, OXPHOS, and FAO. PGC-1α is also extensively regulated by post-translational modifications: PGC-1α is activated *via* phosphorylation by AMPK; its acetylation state is regulated by the counter-balance between SIRT deacetylase and GCN5L1 acetylase. GCN5L1 activation also negatively modulates FAO target genes. Translocation of PPARα between nucleus and IMM affects PPARα activity as a transcription factor. **(A)** In healthy condition. **(B)** Under injury/disease state, impaired PGC-1α leads to defective FAO and is associated with reduced FA catabolism, increased FA pool and TAG accumulation, increased cellular ROS production, and PPARα mitochondrial translocation, which induces PPARα interaction with CypD, mPTP opening, ETC disruption, cytochrome C release, and mitochondrial damage.

Several transcriptional, epigenetic, and post-translational regulators are involved in the crosstalk between peroxisomes, nucleus, and mitochondria to control the expression/functions of FAO enzymes, mitochondrial biogenesis, and energy reprograming in health and disease-stressed states (Stallons et al., 2013; Bhargava and Schnellmann, 2017; Figure 2). These key molecules include the nuclear hormone receptor, peroxisome proliferator-activated receptors (PPARs, PPARα, and PPARγ as examples; Wu et al., 2009; Corrales et al., 2018); PPARγ coactivator 1α (PGC-1α; Weinberg, 2011; Li and Susztak, 2018; Fontecha-Barriuso et al., 2020); the NAD^+^-dependent deacetylases sirtuins (SIRTs; Wakino et al., 2015; Hershberger et al., 2017; Morigi et al., 2018); AMP-activated protein kinase (AMPK); and nuclear respiratory factors 1 and 2 (NRF1 and NRF2; Akhtar and Siragy, 2019).

PGC-1α, the mitochondrial biogenesis master regulator, is predominantly expressed in proximal tubules and interacts directly with multiple transcription factors to integrate upstream signaling events with mitochondrial biogenesis and functional capacity. Downstream transcription factors control all aspects of mitochondrial function, including biogenesis, energy production, dynamics, and protein homeostasis. PGC-1α regulates the expression of NRF1 and NRF2 to increase NAD^+^ biosynthesis (Tran et al., 2016) and activates genes coding for the OXPHOS system (Zoja et al., 2014). In obesity-related nephropathy models, reduced NRF2 along with suppressed expression of the key FAO enzyme long-chain acyl-CoA synthetase-1 (ACSL1) are associated with elevated renal lipid deposition, further supporting the importance of mitochondria in lipid metabolism and energy homeostasis (Chen et al., 2019).

As the PGC-1α/PPARα axis governs transcriptional regulation of FAO, it was proposed as therapeutic target in AKI and CKD (Simon and Hertig, 2015; Stadler et al., 2015). Defects in the FAO pathway, such as reduced expression of CPT-1 (Kang et al., 2015), CrAT (Kruger et al., 2019), and PPARα (Chung et al., 2018) are associated with CKD and renal fibrosis. PPARα heterodimerizes with its obligate partner, the retinoid-X-receptor (RXR), to regulate FAO and energy metabolism. FAs are natural activators of PPARα, and one of PPARα target genes is CD36 (which increases FA uptake; Portilla, 2003). Moreover, impaired expression of PPARα and specific proteins in FAO pathway are associated with lipid accumulation and fibrosis in renal tubular epithelial cells in aging rats (Chung et al., 2018). Interestingly, Jang et al. demonstrated that proximal tubular mitochondrial interaction of PPARα with cyclophilin D (CypD), a component of the IMM structural protein complex mitochondrial permeability transition pore (mPTP), could repress nuclear PPARα activity and negatively modulate FAO in cisplatin-induced AKI (Jang et al., 2020a). Several PPARα agonists have been shown to enhance FAO activity in kidney (Konig et al., 2008; Lakhia et al., 2018).

PGC-1α is extensively regulated by post-translational modifications. AMPK and SIRT positively regulate PGC-1α through phosphorylation or deacetylation, respectively (Jager et al., 2007; Canto and Auwerx, 2009). Interestingly, liver histone demethylase JMJD3 was identified as a gene-specific transcriptional partner of SIRT1 that epigenetically activates mitochondrial β-oxidation during fasting (Seok et al., 2018). The counterpart of JMJD3 in kidney remains to be uncovered. General control of amino acid synthesis-5-like 1 (GCN5L1), a protein acetylase counteracting the function of SIRT3, was recently shown to negatively modulate hepatic FAO enzyme activities *via* acetylation (Thapa et al., 2018). Similarly, GCN5L1-mediated hyper-acetylation and impairment of FAO enzymes might be a key pathogenic event underlying lipid overload-induced kidney injury (Lv et al., 2019).

In summary, the integrated regulation of FA metabolism at the genetic, epigenetic, and protein level is tightly associated with mitochondrial homeostasis. Under injury or disease state, deficiency in PGC-1α and associated transcription factors leads to defective FAO, enlarged FA pool and TAG accumulation, massive ROS production, increased PPARα mitochondrial translocation inducing mPTP opening and loss of mitochondrial membrane potential, cytochrome C release, and mitochondrial damage (Figure 2).

## Dyslipidemia and Cellular Lipotoxicity-Mediated Kidney Injury

Dyslipidemia is an abnormal amount of lipids (e.g., TAG, cholesterol or phospholipids) in the blood. Lipids in excess, which is delivered to organs beyond their energy demands, can be stored mainly as TAG in intracellular lipid droplets (LDs), an ubiquitous organelle that serves as energy stores, dynamic membrane synthesis, and as a hub for further metabolic regulation (Walther et al., 2017). Accumulation of such lipid intermediates or final products in non-adipose tissues, along with the subsequent multi-factorial disturbance of intracellular homeostasis, could result in lipotoxicity of target tissues. Lipotoxicity thus represents a pathologic phenomenon with hallmarks of aberrant lipid accumulation, causing metabolic, inflammatory, oxidative stress in intracellular organelles, and further triggering cell damages (Su et al., 2017; Opazo-Ríos et al., 2020).

Moorhead et al. first hypothesized “lipid nephrotoxicity” in 1982, proposing that dyslipidemia may contribute to the progression of renal dysfunction (Moorhead et al., 1982). This hypothesis had gained supportive evidence in several contexts. For examples, renal lipid accumulation has been shown with high clinical prevalence in patients with CKD, including the insulin resistant obese subjects with diabetic nephropathy (Herman-Edelstein et al., 2014; Escasany et al., 2019; Opazo-Ríos et al., 2020), in nephrotic syndrome (Vaziri, 2016; Agrawal et al., 2017), focal segmental glomerulosclerosis (FSGS; Sasaki et al., 2018), and also as a consequence of acute ischemic renal injury (Zager et al., 2011). Significant alterations in renal lipid metabolism are typified as high TAG, variation in the composition of apolipoproteins and lipids, the accumulation of atherogenic particles VLDL and IDL, and decreased HDL cholesterol (Vaziri, 2006; Stadler et al., 2015; Florens et al., 2016; Kronenberg, 2018; Du and Ruan, 2019; Gai et al., 2019; Thongnak et al., 2020; Jang et al., 2020b). As discussed earlier, systematic lipid metabolism involves multi-organ crosstalk, ultimately also affecting kidney function. Therefore, dyslipidemia and lipid nephrotoxicity could be not only a consequence but also a cause of kidney disease (Florens et al., 2016; Agrawal et al., 2017; Kronenberg, 2018; Czumaj et al., 2019; Nishi et al., 2019).

Excess fat could be derived from either dysfunctional capacity of adipose lipid storage, or from diet-induced hyperlipidemia (high plasma albumin-bound FFAs and cholesterol), or in the condition of renal dysfunction (as commonly exemplified by renal mass reduction in animal model) and defective insulin signaling. Excess kidney ectopic fat deposition and lipid overload in intracellular organelles could lead to ER stress (Zhao et al., 2008), mitochondria dysfunction (Vamecq et al., 2012; Szeto et al., 2016), and lysosomal stress (Yamamoto et al., 2017, 2020). These alterations could change cellular protective mechanisms such as autophagy, mitophagy, lipophagy and contribute to apoptosis and cell damage. These observations thus support the notion of dyslipidemia contributes to the progression of renal injury, and lipid-lowering therapies or shielding mitochondria could provide beneficial effects on lipotoxicity-mediated kidney injury (Izquierdo-Lahuerta et al., 2016; Su et al., 2017).

Dyslipidemia could appear in various forms with different causes and consequences. In lipid-mediated podocyte damage, FFAs and their metabolism affect function and survival of podocytes (Sieber and Jehle, 2014). Dyslipidemia is also a common feature, rather than a complication, of nephrotic syndrome. Excessive urinal protein loss results in hypoproteinemia, in turn leading to low serum oncotic pressure, and even edematous change in severe cases. To rescue the oncotic pressure deficit, the body initiates a reactive hepatic protein synthesis, including lipoproteins (Attman and Alaupovic, 1990; Merscher et al., 2014; Vaziri, 2016; Agrawal et al., 2017). Additionally, reduced plasma levels of lipoprotein lipase results in decreased lipid catabolism. Elevated serum levels of LDL and IDL are filtered through glomeruli and lead to lipiduria, which manifests with fatty casts containing oval fat bodies in the urine sediment (Cavanaugh and Perazella, 2019).

Mutations affecting cholesterol metabolism in the process of lipid trafficking, storage, influx, or efflux, could mediate glomerular injury (Merscher et al., 2014). For example, Tangier disease (OMIM #205400) or DKD caused by mutations in ATP-binding cassette A1 (ABCA1) gene result in reduced HDL in circulation, albuminuria, podocyte phenotype with esterified cholesterol accumulation and dysfunctional mitochondria due to cardiolipin hyperoxidation (Ducasa et al., 2019a,b). For the topics on glomerular diseases-related renal lipotoxicity and mitochondrial dysfunction, please refer to the comprehensive review in the same special issue (Ge et al., 2020).

Furthermore, excess of FFAs leads to TAG accumulation and renal tubular toxicity (Johnson et al., 2005; Scerbo et al., 2017). Increased LCFA-bound albumin induces altered redox balance, high tubular cell apoptosis, and kidney fibrosis (Ruggiero et al., 2014). As lipoprotein abnormalities also correlate with high risk of both cardiovascular and kidney diseases, these modified lipoproteins could be accounted as actual mediators of uremic toxicity (Florens et al., 2016). LDL and oxidized (ox)-LDL uptake by mesangial cells lead to cell proliferation and glomerular matrix expansion, while uptake by PTE results in tubulointerstitial lesions with remarks of heightened expression of extracellular matrix proteins (Nosadini and Tonolo, 2011). HDL is a key player in reverse cholesterol transport to shuttle cholesterol from peripheral cells, such as macrophages, to the liver, therefore relieving the cholesterol burden of these cells. HDL thus exerts its anti-oxidant function through preventing LDL oxidation by ROS and protecting against the adverse effects of ox-LDL on the endothelium. Reduced levels and dysfunction of HDL, which could be due to perturbed HDL proteome composition, are common in CKD patients (Vaziri, 2006; Yamamoto et al., 2012; Agrawal et al., 2017; Kronenberg, 2018; Rysz et al., 2020).

Deficiency of FA metabolism and lipid overload are the main drivers in the progression of both glomerular and tubular kidney diseases. Lipid accumulation, particularly in ischemic proximal tubules, may result in persistent energy depletion with FFA-induced mitochondrial dysfunction, which could play an important role in the AKI to CKD transition (Szeto, 2017). Conversely, mitochondrial protection prevents high-fat diet-induced glomerular and tubular lesions (Szeto et al., 2016).

The pathophysiological changes underlying hyperlipidemia may involve energy shortage from impaired mitochondrial biogenesis or ATP energetics, and systemic oxidative stress due to excessive ROS production accompanied by ER stress and influx of inflammatory cytokines. Without timely intervention, these changes could eventually lead to apoptosis and kidney fibrosis (Agrawal et al., 2017; Du and Ruan, 2019). Less is known about the molecular mechanism of some toxic lipid intermediates (“metabolic poison”) derived from deficiency or decreased expression of FAO-related enzymes in kidney disease development (Stadler et al., 2015; Su et al., 2017); and future research may elucidate this process.

## Targeting Mitochondrial Energy Metabolism and Lipotoxicity in Kidney Diseases

Lipid-lowering therapies in kidney diseases have been studied for many years, although statins is still the first choice of conventional hypolipidemia strategies for its effect on HMGCoA inhibition to block cholesterol synthesis. Cumulative pharmacological efforts have advanced the field to develop classic and novel lipid modifying therapies in kidney diseases, as extensively reviewed recently (Ferro et al., 2018; Sudhakaran et al., 2018; Filippatos et al., 2019; Rosenson et al., 2019; Heine et al., 2020; Opazo-Ríos et al., 2020). These include effective and well-tolerated drugs targeting various lipid synthesis, uptake, trafficking and metabolism pathways. Recent years, compounds that specifically target mitochondria have emerged as promising therapeutic options for patients with renal disease. Here, we discuss molecules targeting mitochondrial lipid metabolism and mitochondrial dysfunction pathways, including pharmacological agents promoting mitochondrial FAO, mitochondrial biogenesis, and ATP synthesis, as well as mitochondrial antioxidants (regulating ROS metabolism) and cardiolipin stabilizers.

### Mitochondrial FAO-Promoting Agents

Carnitine and acetyl-l-carnitine are nonessential nutrients, as kidney of healthy subjects normally produce sufficient carnitine from daily food intake/metabolism and preserve its excretion well. However, carnitine could be used as dietary supplements to help with carnitine shuttle of FAO in conditions of “primary carnitine deficiency” (children with genetic disorder of carnitine transporter OCTN2 encoded by the *SLC22A5* gene; Frigeni et al., 2017) or adults with secondary carnitine deficiencies due to chronic renal failure (Ames, 2010).

PPARα is crucially involved in energy and metabolic homeostasis. Fibrates (fibric acid derivatives, including fenofibrate and the enhanced medication-pemafibrate) are a class of PPARα agonists that lowers blood TAG through decreasing VLDL production by liver and promoting the removal of TAG from blood. Fibrates also moderately increase blood HDL cholesterol. Mechanistically, the PPARα agonists activate PPARα, promote peroxisomal and mitochondrial FAO, initiate cellular cascade to upregulate lipoprotein lipase, and ultimately cause more efficient catabolism of VLDL and TAG (Lakhia et al., 2018; Cheng et al., 2019; Yamashita et al., 2020).

CD36 mediates the internalization of lipids such as LCFAs, oxLDL, and oxidized phospholipid in both proximal tubule cells and podocytes. CD36 signaling is involved in FA-induced glomerular injury (Hua et al., 2015). The ApoA-I mimetic 5A peptide is a CD36 antagonist, which was shown to reduce glomerular injury and tubulointerstitial fibrosis in mouse CKD models of subtotal nephrectomy with angiotensin II infusion or unilateral ureteral obstruction (Souza et al., 2016). 5A peptide was shown to form HDL-like particles to promote ABCA1-dependent cholesterol efflux (Islam et al., 2018) and thus may effectively treat patients with cardiovascular disease.

The herbal alkaloid Berberine (BBR) is used as a supplemental medicine and has shown clinical benefit in reduction of LDL and TAG in diabetic and hypertensive patients (Koppen et al., 2017). BBR has wide spectrum pharmacological effects through its various action of mechanisms such as increasing LDL-receptor mediated hepatic clearance of LDL cholesterol (Wang et al., 2014), protection of lipid-induced apoptosis by promoting FAO in PTEC (Sun et al., 2018), supporting PGC1α-regulated mitochondrial energy homeostasis in CKD model of *db/db* mice and cultured podocytes (Qin et al., 2019a), and podocyte protection *via* inhibition of mitochondrial fission and dysfunction (Qin et al., 2019b).

### Mitochondrial Bioenergetics and Biogenesis-Promoting Agents

Niacin (vitamin B-3) was the first identified lipid-lowering drug in patients at late 1950s and currently used as an adjunct therapy to help the control of cholesterol. Niacin, at pharmacological dose, increases circulating HDL level to improve cholesterol clearance in peripheral tissues and also changes the composition and metabolism of ApoA-I and ApoA-II (Shepherd et al., 1979). The HDL boost effect of niacin is through different molecular mechanisms. First, niacin stabilizes surface ABCA1 expression and ApoA-I lipidation. Second, niacin inhibits surface expression of the hepatic HDL receptor β-ATP synthase, and thus increases HDL blood availability (Zhang et al., 2008). Third, niacin inhibits the hepatic TAG biosynthesis enzyme “diacylglycerol acyltransferase-2 (DGAT2)” to reduce TAG synthesis and leads to the subsequent VLDL/LDL destabilization (Ganji et al., 2004). The mechanisms of DGAT inhibition and TAG metabolism are active research area as more pharmacological drugs designs centering on the two DGAT enzymes (DGAT1 and DGAT2), which apparently have distinct and overlapping functions (Chitraju et al., 2019). Niacin was later found to be an important precursor of cofactor NAD^+^, which promotes SIRT/PGC-1α activity and thus modulates mitochondrial energy homeostasis, biogenesis, and lipid metabolism (Kirkland and Meyer-Ficca, 2018; Romani et al., 2019). Moreover, niacin provides vascular benefits through NAD^+^/SIRT mediated mechanism during endothelial lipotoxicity (Hughes-Large et al., 2014).

The AMPK/SIRT/PGC-1α axis is crucial for mitochondrial biogenesis (Duann and Lin, 2017). Agents modulating this process include metformin. Metformin, the most commonly prescribed drug for the treatment of type 2 diabetes as a glucose-lowering and insulin-sensitizing agent, is a biguanide drug that also actives the energy sensor AMPK. In animal nephropathy models, several pathologies were observed including reduced phosphorylation of acetyl-CoA carboxylase (ACC), a target of AMPK and the major enzyme in the control of FAO rate; decreased expressions of CPT1 and enzymes in mitochondrial biogenesis; and increased lipid accumulation and expression of pro-inflammatory cytokines and tubulointerstitial fibrosis. Metformin reduces renal fibrosis by improving AMPK-mediated phosphorylation of ACC and FA energy metabolism (Lee et al., 2018).

### Mitochondria-Targeted Anti-oxidants

Lipid-mediated mitochondrial oxidative stress is common in many kidney diseases. The selective mitochondria-targeted antioxidants, such as MitoQ and MitoTEMPO, have been developed to mitigate mitochondrial oxidative stress. These small molecule agents could be delivered and concentrated at mitochondria matrix to function as ROS scavenger (Kezic et al., 2016). They are chimeric molecules of a lipophilic cation triphenylphosphonium (TPP+) conjugated with an antioxidant moiety such as ubiquinone (MitoQ; Kelso et al., 2001) or piperidine nitroxides (TEMPOL and TEMPO; Trnka et al., 2008).

MitoTEMPO could be uptaken and accumulated in energized mitochondria matrix several 100-fold to modulate coenzyme Q (CoQ) pool within mitochondria (Trnka et al., 2008). In a diabetic *db/db* mouse model, 7-week of CoQ10 (0.1% in food) oral administration significantly reduced the levels of serum creatinine and blood glucose and albumin-to-creatinine ratio, in accordance with renal morphological restoration (Sun et al., 2019). CoQ10 ameliorates DN-induced mitochondrial dysfunction and oxidative stress through its activation of mitophagy-mediated glomerular mitochondria homeostasis both *in vivo* and *in vitro*. In this study, MitoTEMPO (3 mg/kg/day) restored mitophagy and alleviated kidney dysfunction in glomeruli of *db/db* mice in a similar manner as CoQ10 treatment (Sun et al., 2019). In a mouse sub-total nephrectomy-induced renal fibrosis CKD model, MitoTEMPO rescued impaired renal function and alleviated renal fibrosis by reducing inflammation cytokines, mitochondrial dysfunction, ER stress, and profibrotic factors (Liu et al., 2018).

Additionally, in a clinically relevant murine model of abdominal sepsis (cecal ligation and puncture, CLP), a single delayed high dose of MitoTEMPO (10 mg/kg, given at 6 h post-CLP) could reverse renal mitochondrial dysfunction and attenuated sepsis-induced AKI by 18 h. MitoTEMPO decreased mitochondrial superoxide level, protected ETC respiration, improved renal microcirculation and glomerular filtration rate. Importantly, MitoTEMPO treatment significantly increased 96-h survival rate from 40% in untreated mice to 80% (Patil et al., 2014). The beneficial effect of MitoTEMPO is still under debate as it failed to exert long-term benefits in a later CLP-AKI study (Rademann et al., 2017). However, in a rat puromycin aminonucleoside (PAN)-induced glomerular damage model, a model mimicking children minimal-change nephrotic syndrome (MCNS), a 10-day MitoTEMPO treatment (1 day prior to PAN-injury and continued for 9 additional days) reduced the level of urinary protein, urinary lipid peroxidation and the expression of oxidative stress markers in glomeruli and plasma; although the overall renal function seemed not significantly improved as measure of creatinine clearance (Fujii et al., 2020). In summary, more research is warranted to validate renoprotective effects of MitoTEMPO.

MitoQ is a mitochondria targeted antioxidant of CoQ analogue, which could be accumulated in mitochondria up to 1,000-fold. In a type 1 monogenic diabetes of the young [MODY, the *Ins2Akita* (Akita)] mouse model, oral administration of MitoQ over a 12-week period prevented diabetic nephropathy (Chacko et al., 2010). MitoQ treatment did not alter the glycaemic status of diabetic animals. However, MitoQ significantly decreased urinary albumin levels in diabetic mice. MitoQ offered benefits in prevention of diabetes-induced tubular dysfunction and protection of glomerular function as measured by radioactive tracer clearance capacity. Moreover, MitoQ decreased pathogenic glomerular GBM thickening and reduced interstitial fibrosis through prevention of EMT (epithelial-to-mesenchymal transition) process in Akita mice (Chacko et al., 2010). Recently, in a diabetic *db/db* mouse model, Ward et al. confirmed the renoprotective effects of MitoQ treatment through daily intragastric gavage over a period of 12-week. MitoQ improved renal function, decreased glomerular hyperfiltration, albuminuria, and prevented interstitial fibrosis (Ward et al., 2017). In a mouse ischemia-reperfusion induced AKI (IRI) model, administration of MitoQ prior to the onset of ischemia was shown to reduce oxidative damage and severity of renal IRI (Dare et al., 2015). Despite the great success of mitochondria-targeting antioxidants in preclinical studies, their clinical effects on CKD patients remain to be verified. However, MitoQ supplementation was linked to restoration of endothelial function and reduces aortic arterial stiffness in aging humans, thus offers potential promise in vascular treatment in CKD patients (Rossman et al., 2018).

### Cardiolipin-Targeting Peptides

In mice, a long-term (28 weeks) high fat diet (HFD) caused mitochondrial dysfunction and structural alterations, such as reduction in size and loss of matrix density and IMM cristae, in renal cells including proximal tubular cells, podocytes and glomerular endothelial cells. The mitochondrial injury led to ER stress, lipid droplets accumulation, autophagy, apoptosis, and subsequent inflammation, proteinuria, and fibrosis (Szeto et al., 2016). The mitochondrial injury could be due to loss and/or peroxidation of cardiolipin, the major structural and functional regulator of IMM cristae. Such mitochondrial injury could be prevented with cardiolipin-stabilizing tetrapeptide SS31 (namely, Elamipretide, MTP-13, or Bendavia), which reduces HFD-induced lipid accumulation, toxic ROS production, regulates cytochrome C activity, and restores AMPK signaling (Szeto et al., 2016; Szeto, 2017). The mitochondria protective effect of SS31 after ischemia-AKI prevents prolonged inflammation and arrests CKD transition (Szeto et al., 2017). Elamipretide is on a phase 2a clinical trial in patients with atherosclerotic renal artery stenosis during stent revascularization, with promising results (NCT01755858; Saad et al., 2017) and was shown to improve mitochondria function in the human failing heart (Chatfield et al., 2019). The clinical effects of Elamipretide on kidney disease, however, require further investigations.

## Concluding Remarks

Mitochondria are the “powerhouse” of the high-energy demanding kidney cells. Crosstalk between mitochondria, nucleus, endoplasmic reticulum, and peroxisomes impacts numerous cellular functions. Mitochondrial bioenergetics, adaptation of energy metabolism, and mitochondrial biogenesis during physiology or stress conditions are tightly linked to body lipid homeostasis, as well as health and disease states of kidney. Dysfunctional mitochondria could lead to dyslipidemia, microvasculature damage, inflammation, kidney fibrosis, or even kidney failure. The evolving knowledge of the molecular mechanisms modulating mitochondrial energy homeostasis and lipid metabolism suggest that normalizing renal cell mitochondrial function and energy balance could be an important preventative strategy against dyslipidemia and could provide new drug targets in kidney diseases.

## Author Contributions

All authors contributed to the conception and drafting the work and critically reviewed and revised for the intellectual accuracy of the contents. All authors contributed to the article and approved the submitted version.

### Conflict of Interest

The authors declare that the research was conducted in the absence of any commercial or financial relationships that could be construed as a potential conflict of interest.
